# Database on the mechanical properties of high entropy alloys and complex concentrated alloys

**DOI:** 10.1016/j.dib.2018.11.111

**Published:** 2018-11-28

**Authors:** S. Gorsse, M.H. Nguyen, O.N. Senkov, D.B. Miracle

**Affiliations:** a CNRS, Univ. Bordeaux, ICMCB, UMR 5026, F-33600 Pessac, France; b Bordeaux INP, ENSCBP, F-33600 Pessac, France; c Air Force Research Laboratory, Materials and Manufacturing Directorate, Wright-Patterson AFB, OH 45433, USA

## Abstract

This data article presents the compilation of mechanical properties for 370 high entropy alloys (HEAs) and complex concentrated alloys (CCAs) reported in the period from 2004 to 2016. The data sheet includes alloy composition, type of microstructures, density, hardness, type of tests to measure the room temperature mechanical properties, yield strength, elongation, ultimate strength and Young׳s modulus. For 27 refractory HEAs (RHEAs), the yield stress and elongation are given as a function of the testing temperature. The data are stored in a database provided in Supplementary materials, and for practical use they are tabulated in the present paper. The database was used in recent publications by Miracle and Senkov [1], Gorsse et al. [2] and Senkov et al. [3].

## Specifications table

**Table:** 

Subject area	*Materials Science*
More specific subject area	*High-entropy alloys (HEAs) and complex concentrated alloys (CCAs)*
Type of data	*Table, figure*
How data was acquired	*Compilation of data from available literature. Data extracted from studies on 370 alloys reported in the period from 2004 to 2016.*
Data format	*Analyzed*
Experimental factors	*Data compilation from available literature. Data sheet contains about 81 references.*
Experimental features	*Extensive Data compilation. Alloys’ densities and Young׳s modulus were computed using the rule of mixtures (ROM) for the different reported alloy compositions.*
Data source location	*Data are with the article*
Data accessibility	*Direct submission. Most relevant research article: S. Gorsse, D.B. Miracle, O.N. Senkov, Mapping the world of complex concentrated alloys, Acta Materialia 135 (2017) 177–187**[2]*.

## Value of the data

• The database covers the main mechanical properties of HEAs and CCAs tested under uniaxial loading from published reports since 2004 until end of 2016.
• The database can be used to assess the potential of HEAs and CCAs as possible structural materials.
• The database can be used to represent various property spaces and calculate performance indices.
• The database can enable data mining to extract insights and uncover patterns to guide and accelerate the development of HEAs and CCAs.

## 1 Data

High entropy alloys (HEAs) and complex concentrated alloys (CCAs) represent a new branch of the metallic alloy tree. HEAs are defined as alloys with 5 or more principal elements that have concentrations between 5 and 35 atom percent, promoting the formation of single-phase-disordered solid solutions presumably stabilized by the configurational entropy of mixing. CCAs encompass all alloys, including HEAs, with three or more principal components. CCAs can have single-phase or multi-phase microstructure.

A detailed comparison of CCAs with competing commercial alloys is crucial to identify the most attractive alloys for structural applications and guide future studies [1], [2], [3]. The relative merits of these new alloys depend on combinations of properties specific to the applications and loading conditions. Thus, this data article is a compilation of the density and mechanical properties of CCAs published in the literature since 2004, allowing the performance indices for lighter, stronger and stiffer structures to be evaluated for different loading conditions [2]. The data are stored in a database and tabulated in the present article.

## 2 Experimental design, materials and methods

**Fig. 1:**
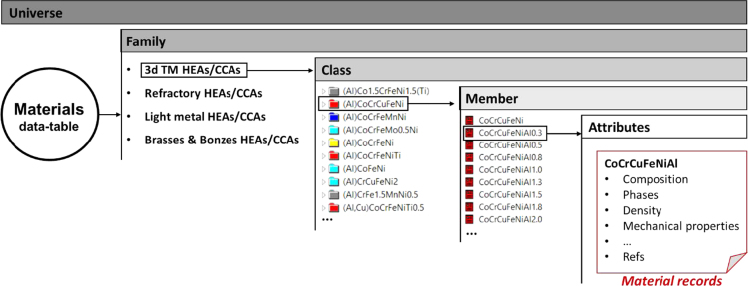
Tree-like classification of the HEAs/CCAs database.

The database has a tree-like classification (Fig. 1) which includes four different families: 3d transition metal (3d TM), refractory metal (RHEAs and RCCAs), light metal family, and bronzes and brasses HEAs/CCAs. Each family is expanded in classes (a class is a unique combination of principal elements), and each class contains members having variations in principal element concentrations. Each member is characterized by a set of attributes which includes: alloy composition, phase content, density, hardness (Vickers), type of mechanical test (tension or compression), yield strength, ultimate strength, elongation, and Young's modulus. A listing of these entries makes up a material record. The database was used by Gorsse et al. [2] with Cambridge Education Software (CES) enabling users to (i) browse the materials data, (ii) search and filter to narrow down the set of materials using given parameters (e.g. alloy composition that contains a specific chemical element), (iii) represent material property maps by plotting any properties or combination of properties against any other property, and (iv) select materials using performance indices as defined by M. F. Ashby.

**Fig. 2:**
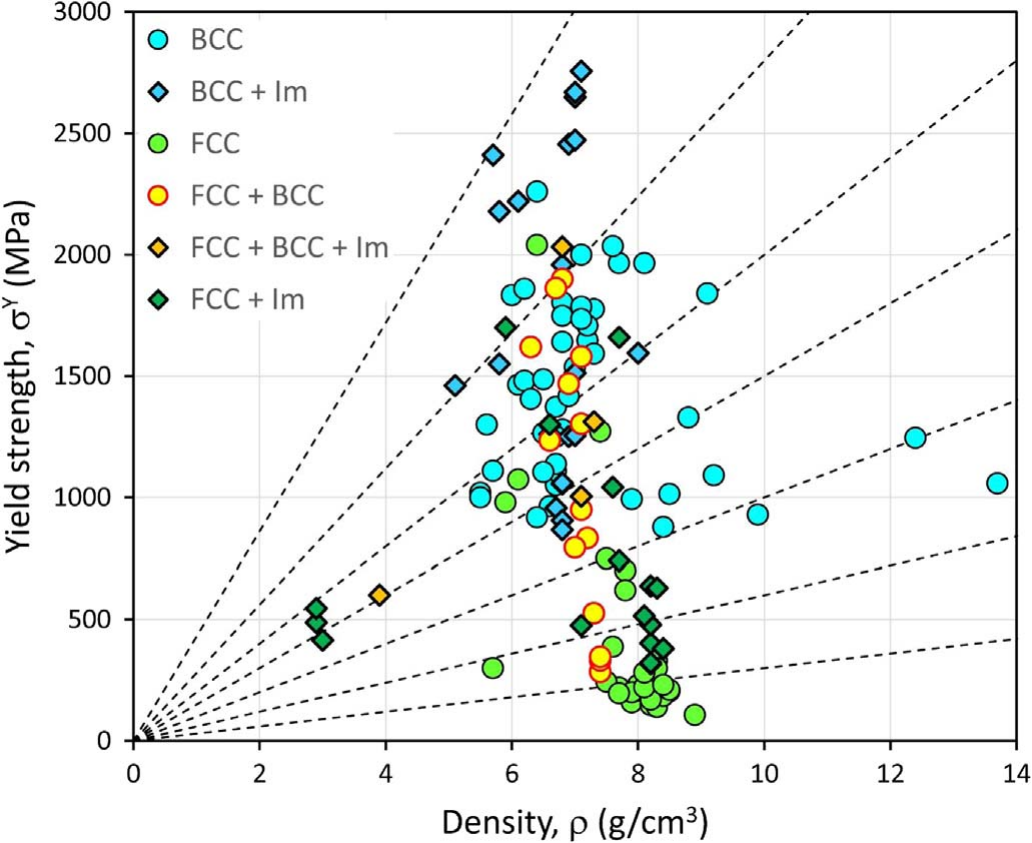
Materials property space for room temperature yield strength vs density of HEAs and CCAs. Alloy members have been colored to identify crystal structure (Im stands for intermetallic). The lines give performance index for uniaxial loading (corresponding to the material index *σ^Y^*/*ρ* where *σ^Y^* and *ρ* are the yield strength and the density, respectively).

A representation of the data is illustrated in Fig. 2 where the room temperature yield strength is plotted against the density for CCAs.

Since this work reflects the state of the art of the field of HEAs and CCAs, the properties are not equally populated for every alloy due to the lack of literature data. The density of the alloy was estimated using the rule of mixtures (ROM): $\rho =\sum x_{i}M_{i}/\sum x_{i}V_{i}$ where $x_{i}$, $M_{i}$ and $V_{i}$ are the atomic fraction, molar mass and molar volume of the element *i*. When not experimentally measured, the Young׳s modulus was estimated using ROM for single phase solid solutions only: $E=\sum x_{i}E_{i}$ where $E_{i}$ is the Young modulus of the alloy element *i*.

For practical use by all, the data are also given in the present article using Tables and shared on Google Drive via the following link: https://docs.google.com/spreadsheets/d/1hLiqmlysSKK7Ubv362v8fasoh8-W17V7zqNzRfSoilw/edit?usp=sharing. The main entries for 370 alloy compositions are listed at room temperature in Table 1, while Table 2 shows the temperature dependence of the mechanical properties for 27 HEAs/CCAs. Each row in Table 1 corresponds to one mechanical test for an alloy composition in an experimentally characterized metallurgical condition.

**Table 1:** HEAs and CCAs for which mechanical tests are reported in literature. *ρ* represents the density, HV is the hardness in Vickers, *σ^Y^* is the Yield strength, *σ^max^* is the ultimate strength, *ε* is the elongation and *E* is the Young's modulus. Parentheses indicate values estimated using ROM. In the column “Type of tests”, C and T stands for compression and tension. Im stands for Intermetallic. Each row represents the result of a test on a specific alloy composition.

**Composition (atomic)**	**Ref.**	**Type of phases**	***ρ* (g/cm**^**3**^**)**	**HV**	**Type of tests**	***σ**^**y**^***(MPa)**	***σ**^**max**^***(MPa)**	***ε* (%)**	***E* (GPa)**
**3d TM HEAs and CCAs in the Al-Co-Cr-Fe-Mn-Ni system and derivates**									
CoFeNi	[4]	FCC	(8.5)	125	C	204			(207)
CoFeNi	[4]	FCC	(8.5)	125	C	209			(207)
CoFeNi	[5]	FCC	(8.5)		T	211	513	31	(207)
CoFeNiSi0.25	[4]	FCC	(7.7)	149	C	196			(194)
CoFeNiSi0.5	[4]	FCC + Im	(7.1)	287	C	476			
CoFeNiSi0.75	[4]	FCC + Im	(6.6)	570	C	1301			
Al0.25CoFeNi	[4]	FCC	(7.9)	138	C	158			(196)
Al0.5CoFeNi	[4]	FCC + BCC	(7.4)	212	C	346			(187)
Al0.75CoFeNi	[4]	FCC + BCC	(7.0)	385	C	794			(179)
CoCrFeNi	[6]	FCC	(8.2)		T	148	413	48	(225)
CoCrFeNi	[7]	FCC	(8.2)	116					(225)
CoCrFeNi	[7]	FCC	(8.2)	113					(225)
CoCrFeMo0.5Ni	[8]	FCC + Im	(8.5)	210					
CoCrFeNb0.103Ni	[6]	FCC + Im	(8.2)		T	318	622	19	
CoCrFeNb0.155Ni	[6]	FCC + Im	(8.2)		T	322	744	23	
CoCrFeNb0.206Ni	[6]	FCC + Im	(8.2)		T	403	807	9	
CoCrFeNb0.309Ni	[6]	FCC + Im	(8.2)		T	479	879	4	
CoCrFeNb0.412Ni	[6]	FCC + Im	(8.2)		T	638	1004	1	
CoCrFeNiTi	[9]	FCC	(7.2)		C		2020	9	135 (203)
Co1.5CrFeNi1.5Ti0.5	[10]	FCC	(7.8)	509					(211)
Co1.5CrFeNi1.5Ti	[10]	FCC + Im	(7.4)	654					
Al0.25CoCrFeNi	[7]	FCC	(7.7)	110					(216)
Al0.25CoCrFeNi	[7]	FCC	(7.7)	113					(216)
Al0.375CoCrFeNi	[7]	FCC	(7.5)	131					(211)
Al0.375CoCrFeNi	[7]	FCC	(7.5)	196					(211)
Al0.5CoCrFeNi	[7]	FCC + BCC	(7.3)	159					(208)
Al0.5CoCrFeNi	[7]	FCC + BCC	(7.3)	209					(208)
Al0.7Co0.3CrFeNi	[11]	FCC + BCC + B2	(6.8)	624	C	2033	2635	8	
Al0.75CoCrFeNi	[7]	FCC + BCC	(7.0)	388					(200)
Al0.75CoCrFeNi	[7]	FCC + BCC	(7.0)	280					(200)
Al0.875CoCrFeNi	[12]	FCC + BCC	(6.9)						(197)
Al0.875CoCrFeNi	[7]	BCC	(6.9)	538					(197)
Al0.875CoCrFeNi	[7]	FCC + BCC	(6.9)	361					(197)
AlCoCrFeNi	[7]	BCC	(6.7)	484					(194)
AlCoCrFeNi	[7]	FCC + BCC	(6.7)	433					(194)
AlCoCrFeNi	[13]	BCC	(6.7)	395					(194)
AlCoCrFeNi	[14]	BCC	(6.7)		C	1251	2004	33	(194)
AlCoCrFeNi	[15]	BCC	(6.7)		C	1051			(194)
AlCoCrFeNi	[16]	BCC	(6.7)		C	1110			(194)
AlCoCrFeNi	[17]	BCC	(6.7)		C	1138			125 (194)
AlCoCrFeNi	[18]	BCC	(6.7)		C	1138		11	125 (194)
AlCoCrFeNi	[19]	BCC	(6.7)		C	1051			(194)
AlCoCrFeNi	[20]	BCC	(6.7)	520	C	1373	3531	25	(194)
Al1.25CoCrFeNi	[7]	BCC	(6.5)	487					(188)
Al1.25CoCrFeNi	[7]	BCC	(6.5)	499					(188)
Al1.5CoCrFeNi	[7]	BCC	(6.2)	484					(183)
Al1.5CoCrFeNi	[7]	BCC	(6.2)	517					(183)
Al1.5CoCrFeNi	[13]	BCC	(6.2)	402					(183)
Al2CoCrFeNi	[7]	BCC	(5.9)	509					(173)
Al2CoCrFeNi	[7]	BCC	(5.9)	512					(173)
Al2CoCrFeNi	[13]	BCC	(5.9)	432					(173)
Al2.5CoCrFeNi	[13]	BCC	(5.6)	487					(165)
Al3CoCrFeNi	[13]	BCC	(5.3)	506					(158)
AlC0.1CoCrFeNi	[18]	BCC + Im	(6.7)		C	957	2550	11	213
AlC0.2CoCrFeNi	[18]	BCC + Im	(6.8)		C	906	2386	9	151
AlC0.3CoCrFeNi	[18]	BCC + Im	(6.8)		C	867	2178	8	137
AlC0.4CoCrFeNi	[18]	BCC + Im	(6.8)		C	1056	2375	7	156
AlC0.5CoCrFeNi	[18]	BCC + Im	(6.8)		C	1060	2250	6	181
AlCCoCrFeNi	[18]	BCC + Im	(6.9)		C	1251	2166	7	75
AlC1.5CoCrFeNi	[18]	BCC + Im	(7.0)		C	1255	2083	6	73
Al0.5CoCrFeMo0.5Ni	[8]	FCC + Im	(7.7)	425					
AlCo0.5CrFeMo0.5Ni	[21]	BCC + Im	(7.0)	801					
AlCoCrFe0.5Mo0.5Ni	[22]	BCC + Im	(7.0)	755					
AlCoCrFe0.6Mo0.5Ni	[22]	BCC + Im	(7.1)	754					
AlCoCrFeMo0.1Ni	[19]	BCC	(6.8)		C	1804	2280	9	(196)
AlCoCrFeMo0.2Ni	[19]	BCC + Im	(6.9)		C	2456	2953	3	
AlCoCrFeMo0.3Ni	[19]	BCC + Im	(7.0)		C	2649	3208	3	
AlCoCrFeMo0.4Ni	[19]	BCC + Im	(7.0)		C	2670	3161	3	
AlCoCrFeMo0.5Ni0.5	[23]	BCC + Im	(7.0)	708					
AlCoCrFeMo0.5Ni	[19]	BCC + Im	(7.1)		C	2757	3036	3	
AlCoCrFeMo0.5Ni	[21]	BCC + Im	(7.1)	796					
AlCoCrFeMo0.5Ni	[8]	BCC + Im	(7.1)	715					
AlCoCrFeMo0.5Ni	[23]	BCC + Im	(7.1)	730					
AlCoCrFeMo0.5Ni1.5	[23]	FCC + BCC + Im	(7.2)	586					
AlCoCrFeMo0.5Ni2	[23]	FCC + BCC + Im	(7.4)	395					
AlCo1.5CrFeMo0.5Ni	[21]	BCC + Im	(7.2)	741					
AlCo2CrFeMo0.5Ni	[21]	FCC + BCC + Im	(7.3)	586					
AlCoCrFe1.5Mo0.5Ni	[22]	BCC + Im	(7.2)	635					
AlCoCrFe2Mo0.5Ni	[22]	BCC + Im	(7.2)	639					
Al1.5CoCrFeMo0.5Ni	[8]	BCC + Im	(6.6)	655					
Al2CoCrFeMo0.5Ni	[8]	BCC	(6.3)	605					(185)
AlCoCrFeNb0.1Ni	[20]	BCC	(6.8)	569	C	1641	3285	17	(192)
AlCoCrFeNb0.25Ni	[20]	BCC + Im	(6.8)	668	C	1959	3008	11	
AlCoCrFeNb0.5Ni	[20]	BCC + Im	(7.0)	747	C	2473	3170	4	
AlCoCrFeNb0.75Ni	[20]	BCC + Im	(7.0)						
AlCoCrFeNiSi0.2	[24]	BCC	(6.5)		C	1265	2173	14	(188)
AlCoCrFeNiSi0.4	[24]	BCC	(6.2)		C	1481	2444	13	(183)
AlCoCrFeNiSi0.6	[24]	BCC	(6.0)		C	1834	2195	3	(178)
AlCoCrFeNiSi0.8	[24]	BCC + Im	(5.8)		C	2179	2664	2	
AlCoCrFeNiSi	[24]	BCC	(5.7)		C	1110			(169)
AlCoCrFeNiSi	[24]	BCC + Im	(5.7)		C	2411	2950	1	
Al0.2Co1.5CrFeNi1.5Ti0.5	[10]	FCC	(7.6)	487					(206)
Al0.2Co1.5CrFeNi1.5Ti	[10]	FCC + Im	(7.2)	717					
Al0.5CoCrFeNiTi	[9]	BCC + Im	(6.6)		C		1600	10	107
AlCoCrFeNiTi0.5	[25]	FCC	(6.4)	178	C	2040	3135	24	72 (187)
AlCoCrFeNiTi0.5	[26]	BCC	(6.4)	178	C	2260	3140	23	178 (187)
AlCoCrFeNiTi	[26]	BCC	(6.2)		C	1860	2580	9	90 (181)
AlCoCrFeNiTi	[9]	BCC + Im	(6.2)		C		2280	6	148
AlCoCrFeNiTi1.5	[26]	BCC + Im	(6.1)		C	2220	2720	5	160
Al1.5CoCrFeNiTi	[9]	BCC	(5.9)		C		2110	10	133 (172)
Al2CoCrFeNiTi	[9]	BCC	(5.6)	643	C		1030	5	94 (165)
AlCoCrFeNiTiVZr	[27]		(6.3)	780					
CoCrFeMnNi	[28]	FCC	(8.0)	176	T	208		62	(219)
CoCrFeMnNi	[29]	FCC	(8.0)	144	C	230		75	(219)
CoCrFeMnNiV0.25	[29]	FCC	(7.9)	151	C	200		75	(215)
CoCrFeMnNiV0.5	[29]	FCC	(7.8)	186	C	620		75	(211)
CoCrFeMnNiV0.75	[29]	FCC + Im	(7.7)	342	C	740	1325	8	
CoCrFeMnNiV1.0	[29]	FCC + Im	(7.7)	650	C	1660	1845	< 1	
Al0.10CoCrFeMnNi	[28]	FCC	(7.9)	180					(216)
Al0.20CoCrFeMnNi	[28]	FCC	(7.7)	171	T	220		56	(214)
Al0.38CoCrFeMnNi	[28]	FCC	(7.5)	182	T	244		45	(209)
Al0.43CoCrFeMnNi	[28]	FCC + BCC	(7.4)	183	T	285		35	(208)
Al0.49CoCrFeMnNi	[28]	FCC + BCC	(7.4)	220	T	331		29	(206)
Al0.56CoCrFeMnNi	[28]	FCC + BCC	(7.3)	278	T	526		16	(204)
Al0.62CoCrFeMnNi	[28]	FCC + BCC	(7.2)	405	T	833		5	(203)
Al0.68CoCrFeMnNi	[28]	FCC + BCC	(7.2)	486					(202)
Al0.75CoCrFeMnNi	[28]	FCC + BCC	(7.1)	530					(200)
Al0.81CoCrFeMnNi	[28]	FCC + BCC	(7.0)	539					(199)
Al0.88CoCrFeMnNi	[28]	FCC + BCC	(7.0)	533					(197)
Al0.95CoCrFeMnNi	[28]	FCC + BCC	(6.9)	535					(196)
Al1.25CoCrFeMnNi	[28]	BCC	(6.6)	539					(190)
CoCrNi	[5]	FCC	(8.3)		T	300	860	60	(229)
CoMnNi	[5]	FCC	(8.4)		T	231	653	38	(202)
FeMnNi	[5]	FCC	(8.1)		T	221	602	36	(203)
CoCrFeNi	[5]	FCC	(8.2)		T	274	708	39	(225)
CoCrMnNi	[5]	FCC	(8.1)		T	282	694	44	(222)
CoFeMnNi	[5]	FCC	(8.2)		T	170	550	41	(205)
Al0.5CrFe1.5MnNi0.5	[30]	BCC	(7.0)	396					(206)
Al0.3CrFe1.5MnNi0.5	[30]	FCC + BCC	(7.2)	297					(213)
AlCoCrFeMo0.5	[23]	BCC + Im	(6.8)	857					
AlCrFeNi	[31]	BCC	(6.3)	472	C	1406	2927	29	(190)
AlCrFeNiMo0.2	[31]	BCC	(6.5)	549	C	1487	3222	29	(197)
AlCrFeNiMo0.5	[31]	BCC	(6.8)	622	C	1749	2644	13	(205)
AlCrFeNiMo0.8	[31]	BCC + Im	(7.0)	854	C	1513	1513	< 1	
AlCrFeNiMo	[31]	BCC + Im	(7.2)	905					
**3d TM HEAS and CCAs in the Al-Co-Cr-Cu-Fe-Mn-Ni system and derivates**									
CoCrCuFe	[32]	FCC	(8.2)	134					(206)
Al0.3CoCrCuFe	[32]	FCC	(7.7)	180					(194)
Al0.5CoCrCuFe	[32]	FCC	(7.4)	207					(187)
Al0.8CoCrCuFe	[32]	FCC + BCC	(7.0)	271					(177)
AlCoCrCuFe	[32]	FCC + BCC	(6.8)	407					(172)
Al1.3CoCrCuFe	[32]	FCC + BCC	(6.5)	476					(165)
Al1.5CoCrCuFe	[32]	FCC + BCC	(6.3)	510					(167)
Al1.8CoCrCuFe	[32]	FCC + BCC	(6.0)	557					(155)
Al2.0CoCrCuFe	[32]	FCC + BCC	(5.9)	567					(152)
Al2.3CoCrCuFe	[32]	FCC + BCC	(5.7)	603					(147)
Al2.5CoCrCuFe	[32]	FCC + BCC	(5.6)	624					(144)
Al2.8CoCrCuFe	[32]	BCC	(5.5)	657					(140)
Al3.0CoCrCuFe	[32]	BCC	(5.4)	644					(138)
CoCrCu0.5FeNi	[33]	FCC	(8.3)	172					(214)
CoCrCuFeNi	[34]	FCC	(8.3)	132	C	230			56 (206)
CoCrCuFeNi	[45]	FCC	(8.3)	286	C	230	888	51	56 (206)
CoCrCuFeNi	[13]	FCC	(8.3)	286					(206)
CoCrCuFeNiTi0.5	[25]	FCC	(7.8)		C	700	1650	29	93 (198)
CoCrCuFeNiTi0.5	[35]	FCC	(7.8)		C	700	1650	22	99 (198)
CoCrCuFeNiTi0.8	[35]	FCC + Im	(7.6)		C	1042	1848	3	128
CoCrCuFeNiTi	[35]	FCC	(7.4)		C	1272	1272	2	77 (191)
Al0.25CoCrCu0.5FeNiTi0.5	[25]	FCC	(7.4)						(198)
Al0.25CoCrCu0.75FeNiTi0.5	[25]	FCC	(7.5)		C	750	1970	39	103 (195)
Al0.3CoCrCuFeNi	[34]	FCC	(7.9)	180					(198)
Al0.5CoCrCuFeNi	[34]	FCC	(7.6)	210	C	388			(193)
Al0.5CoCrCuFeNi	[36]		(7.6)	300					(193)
Al0.5CoCrCuFeNi	[37]	FCC	(7.6)	225					(193)
Al0.5CoCrCuFeNi	[38]	FCC	(7.6)	215					(193)
Al0.8CoCrCuFeNi	[21]	FCC + BCC	(7.3)	270					(187)
Al0.8CoCrCuFeNi	[34]	FCC	(7.3)	270					(187)
AlCoCrCuFeNi	[34]	FCC + BCC	(7.1)	406	C	950			(183)
AlCoCrCuFeNi	[39]	FCC + BCC	(7.1)	472					(184)
AlCoCrCuFeNi	[40]	FCC + BCC	(7.1)		C	1303		24	(183)
AlCoCrCuFeMnNi	[40]	FCC + BCC + Im	(7.1)		C	1005		15	
AlCoCrCuFeNiTi	[40]	FCC + BCC	(6.6)		C	1234		9	(174)
AlCoCrCuFeNiV	[40]	FCC + BCC	(6.9)		C	1469		16	(175)
Al1.3CoCrCuFeNi	[34]	FCC + BCC	(6.8)	470					(178)
Al1.5CoCrCuFeNi	[34]	FCC + BCC	(6.6)	506					133 (174)
Al1.8CoCrCuFeNi	[34]	FCC + BCC	(6.4)	650					(170)
Al2CoCrCuFeNi	[34]	FCC + BCC	(6.3)	560	C	1620			(167)
Al2.3CoCrCuFeNi	[34]	FCC + BCC	(6.1)	600					(163)
Al2.5CoCrCuFeNi	[34]	FCC + BCC	(6.0)	620					(161)
Al2.8CoCrCuFeNi	[34]	BCC	(5.8)	650					(157)
Al3CoCrCuFeNi	[41]	BCC	(5.7)	640					(153)
Al0.5B0.2CoCrCuFeNi	[36]		(7.7)	415					
Al0.5B0.6CoCrCuFeNi	[36]		(7.7)	505					
Al0.5BCoCrCuFeNi	[36]		(7.8)	736					
Al0.5CoCrCu0.5FeNiTi0.5	[25]	FCC + BCC	(7.1)		C	1580	2389	17	161 (192)
Al0.5CoCrCuFeNiTi0.2	[37]	FCC	(7.5)	272					(191)
Al0.5CoCrCuFeNiTi0.4	[37]	FCC	(7.3)	321					(188)
Al0.5CoCrCuFeNiTi0.6	[37]	FCC + BCC	(7.2)	458					(186)
Al0.5CoCrCuFeNiTi0.8	[37]	FCC + BCC	(7.1)	590					(184)
Al0.5CoCrCuFeNiTi	[37]	FCC + BCC + Im	(7.0)	636					
Al0.5CoCrCuFeNiTi1.2	[37]	FCC + BCC + Im	(6.9)	646					
Al0.5CoCrCuFeNiTi1.4	[37]	FCC + BCC + Im	(6.8)	664					
Al0.5CoCrCuFeNiTi1.6	[37]	FCC + BCC + Im	(6.7)	657					
Al0.5CoCrCuFeNiTi1.8	[37]	FCC + BCC + Im	(6.6)	667					
Al0.5CoCrCuFeNiTi2	[37]	FCC + BCC + Im	(6.5)	696					
Al0.5CoCrCuFeNiV0.2	[38]	FCC	(7.6)	204					(191)
Al0.5CoCrCuFeNiV0.4	[38]	FCC + BCC	(7.5)	231					(189)
Al0.5CoCrCuFeNiV0.6	[38]	FCC + BCC + Im	(7.5)	328					
Al0.5CoCrCuFeNiV0.8	[38]	FCC + BCC + Im	(7.4)	447					
Al0.5CoCrCuFeNiV1.0	[38]	FCC + BCC + Im	(7.4)	639					
Al0.5CoCrCuFeNiV1.2	[38]	BCC	(7.3)	579					(182)
Al0.5CoCrCuFeNiV1.4	[38]	BCC	(7.3)	577					(180)
Al0.5CoCrCuFeNiV1.6	[38]	BCC	(7.2)	594					(179)
Al0.5CoCrCuFeNiV1.8	[38]	BCC	(7.2)	597					(177)
Al0.5CoCrCuFeNiV2.0	[38]	BCC	(7.2)	587					(176)
Al0.75CoCrCu0.25FeNiTi0.5	[25]	FCC + BCC	(6.8)		C	1900	2697	12	164 (189)
AlCoCrCuNiTi	[42]	BCC	(6.4)		C		1495	8	36 (167)
AlCoCrCuNiTiY0.5	[42]	Im	(6.1)		C		1025	3	36
AlCoCrCuNiTiY0.8	[42]	Im	(5.9)		C		1325	5	38
AlCoCrCuNiTiY	[42]	Im	(5.8)		C		1192	4	37
AlCoFeNi	[4]	BCC	(6.6)	456	C	964			(173)
AlCoFeNiTiVZr	[27]	BCC	(6.2)	790					(143)
CoCuFeNi	[43]	FCC	(8.6)		T		480	15	(188)
CoCuFeNiSn0.02	[43]	FCC	(8.6)		T		548	17	(187)
CoCuFeNiSn0.04	[43]	FCC + Im	(8.6)		T		594	18	
CoCuFeNiSn0.05	[43]	FCC + Im	(8.6)		T		615	20	
CoCuFeNiSn0.07	[43]	FCC + Im	(8.6)		T		632	19	
CoCuFeNiSn0.1	[43]	FCC + Im	(8.6)		T		602	5	
CoCuFeNiSn0.2	[43]	FCC + Im	(8.5)		T		261	2	
CoCuFeNiSn0.5	[43]	FCC + Im	(8.3)						
AlCoCuFeNi	[39]	FCC + BCC	(7.0)	536					(164)
AlCoCuFeNbNi	[39]	Im	(7.4)	578					
AlCoCuFeNiSi	[39]	FCC + BCC	(5.9)	682					(145)
AlCoCuFeNiTi	[39]	FCC + BCC	(6.5)	626					(156)
AlCoCuFeNiZr	[39]	FCC + BCC + Im	(6.9)	472					
CoCuFeMnNi	[44]	FCC	(8.4)	208	T		478	14	(190)
CoCuFeMnNiSn0.03	[44]	FCC	(8.4)	192	T		465	18	
CoCuFeMnNiSn0.05	[44]	FCC + Im	(8.4)	205	T		475	12	
CoCuFeMnNiSn0.08	[44]	FCC + Im	(8.3)	219	T		425	7	
CoCuFeMnNiSn0.10	[44]	FCC + Im	(8.3)	253	T		470	6	
CoCuFeMnNiSn0.20	[44]	FCC + Im	(8.3)	319	T		368	2	
CrCuFeMnNi	[13]	FCC + BCC	(8.1)	296					(204)
CrCuFeMoNi	[13]	FCC	(8.7)	263					(230)
AlCrCuFeNi0.6	[45]	FCC + BCC	(6.6)	496					(176)
AlCrCuFeNi0.8	[45]	FCC + BCC	(6.7)	486					(177)
AlCrCuFeNi	[45]	FCC + BCC	(6.8)	495					(178)
AlCrCuFeNi1.2	[45]	FCC + BCC	(6.8)	407					(179)
AlCrCuFeNi1.4	[45]	FCC + BCC	(6.9)	367					(180)
AlCrCuFeNi2	[46]	FCC + BCC	(7.1)						(182)
AlCrCuFeNiTi	[47]	BCC + Im	(6.3)		C		1219		
Al0.2CrCuFeNi2	[46]	FCC	(8.0)						(199)
Al0.4CrCuFeNi2	[46]	FCC	(7.8)						(194)
Al0.6CrCuFeNi2	[46]	FCC	(7.5)						(190)
Al0.8CrCuFeNi2	[46]	FCC	(7.3)						(186)
Al1.2CrCuFeNi2	[46]	FCC + BCC	(6.9)						(178)
AlCrCuFeNi	[13]	FCC + BCC	(6.8)	342					(178)
Al1.125CuFe0.75NiTi1.125	[48]	FCC	(5.9)	516	C	980	1326	7	145 (140)
Al22.5Cu20Fe15Ni20Ti22.5	[48]	FCC	(5.9)	516	C	980	1326	7	145 (140)
AlCuFeNiTi	[48]	FCC	(6.1)	516	C	1074	1617	8	146 (145)
AlCuNiTi	[48]	FCC	(5.7)	537	C	300	536	< 1	108 (129)
**Light metal base HEAs and CCAs**									
AlLi0.5MgSn0.2Zn0.5	[49]	FCC + Im	(2.9)		C	546	546		
AlLiMg0.5ScTi1.5	[50]	FCC + HCP	(2.7)	591					(69)
AlLiMgSnZn	[49]	FCC + HCP + Im	(3.9)		C	600	615	1	
Al8Li0.5Mg0.5Sn0.5Zn0.5	[49]	FCC + Im	(3.0)		C	415	836	16	
Al8Cu0.5Li0.5Mg0.5Zn0.5	[49]	FCC + Im	(2,9)		C	488	879	17	
AlCu0.2Li0.5MgZn0.5	[49]	Im	(2.7)						
AlCu0.5Li0.5MgSn0.2	[49]	Im	(3.0)						
**Refractory metal base HEAs and CCAs**									
AlCr0.5NbTiV	[51]	BCC	(5.6)		C	1300	1430	< 1	(124)
AlCrNbTiV	[51]	BCC + Im	(5.8)		C	1550	1570	< 1	
AlCr1.5NbTiV	[51]	FCC + Im	(5.9)		C	1700	1700	< 1	
Al0.4Hf0.6NbTaTiZr	[52]	BCC	(9.1)	500	C	1841	2269	10	(110)
Al0.3HfNbTaTiZr	[53]	BCC	9.5 (9.6)	353	C	1188		50	63 (108)
Al0.5HfNbTaTiZr	[53]	BCC	9.34 (9.3)	396	C	1302		46	97 (107)
Al0.75HfNbTaTiZr	[53]	BCC	9.3 (9.1)	427	C	1415		30	102 (105)
AlMo0.5NbTa0.5TiZr	[52]	BCC	(7.1)	591	C	2000	2368	10	(123)
Al0.25MoNbTiV	[54]	BCC	(7.1)	460	C	1250		13	(164)
Al0.5MoNbTiV	[54]	BCC	(6.8)	487	C	1625		11	(158)
Al0.75MoNbTiV	[54]	BCC	(6.6)	517	C	1260		8	(154)
AlMoNbTiV	[54]	BCC	(6.4)	537	C	1375		3	(150)
Al0.25NbTaTiV	[55]	BCC	(8.8)		C	1330			92 (130)
Al0.5NbTaTiV	[55]	BCC	(8.5)		C	1014			97 (127)
AlNbTaTiV	[55]	BCC	(7.9)		C	993			101 (121)
Al0.3NbTa0.8Ti1.4V0.2Zr1.3	[52]	BCC	(7.7)	500	C	1965	2061	5	(110)
Al0.5NbTa0.8Ti1.5V0.2Zr	[52]	BCC	(7.6)	530	C	2035	2105	5	(111)
Al0.3NbTaTi1.4Zr1.3	[52]	BCC	(8.1)	490	C	1965	2054	5	(113)
AlNb1.5Ta0.5Ti1.5Zr0.5	[52]	BCC	(6.8)	408	C	1280	1367	4	(106)
AlNbTiV	[56]	BCC	(5.5)	448	C	1020	1318	5	(105)
AlNbTiV	[51]	BCC	(5.5)		C	1000	1280	5	(105)
CrHfNbTiZr	[57]	BCC + lm	(8.2)	464	C	1375	2130	3	112
CrMo0.5NbTa0.5TiZr	[58]	BCC + Im	(8.0)	540	C	1595	2046	5	
CrNbTiVZr	[59]	BCC + Im	(6.6)	482	C	1298		3	
CrNbTiZr	[59]	BCC + Im	(6.6)	418	C	1260		6	
FeMoNiTiVZr	[27]	BCC + Im	(7.1)	740					
Hf0.5Mo0.5NbTiZr	[60]	BCC + Im	(7.9)	400	C	1178		25	
Hf0.5Mo0.5NbSi0.1TiZr	[60]	BCC + Im	(7.7)	442	C	1365		28	
Hf0.5Mo0.5NbSi0.3TiZr	[60]	BCC + Im	(7.5)	494	C	1428		23	
Hf0.5Mo0.5NbSi0.5TiZr	[60]	BCC + Im	(7.2)	524	C	1605		23	
Hf0.5Mo0.5NbSi0.7TiZr	[60]	BCC + Im	(7.0)	580	C	1604		12	
Hf0.5Mo0.5NbSi0.9TiZr	[60]	BCC + Im	(6.8)	640	C	1677		9	
Hf0.5Mo0.5NbTiZrC0.1	[61]	BCC + lm	(7.8)		C	1183	2139	38	
Hf0.5Mo0.5NbTiZrC0.3	[61]	BCC + lm	(7.7)		C	1201	1965	33	
HfMo0.25NbTaTiZr	[62]	BCC	9.9 (9.9)	395	C	1112		50	96 (121)
HfMo0.5NbTaTiZr	[62]	BCC	10.0 (9.9)	480	C	1317		50	102 (130)
HfMo0.75NbTaTiZr	[62]	BCC	10.0 (9.9)	492	C	1373		50	109 (139)
HfMoNbTaTiZr	[63]	BCC	10.0 (10.0)	505	C	1512		12	(147)
HfMoNbTaTiZr	[62]	BCC	10.0 (9.9)	505	C	1512		12	115 (147)
HfMoTaTiZr	[63]	BCC	10.2 (10.2)	542	C	1600		4	(155)
HfMoNbZrTi	[64]	BCC	(8.7)		C	1803	1719	10	(139)
HfNbSi0.5TiV	[65]	BCC + lm	8.6 (7.8)	490	C	1399	1608	11	
HfNbSi0.5TiVZr	[66]	BCC + lm	7.8 (7.5)	464	C	1540	1643	17	
HfNbTaZr	[67]	BCC	(11.1)	365	C	1315			(109)
Hf0.5Nb0.5Ta0.5Ti1.5Zr	[68]	BCC	8.1 (8.2)	301	T	903	990	19	(107)
HfNbTaTiZr	[62]	BCC	9.9 (9.9)	335	C	1015		50	85 (111)
HfNbTaTiZr	[53]	BCC	9.7 (9.9)	295	C	1073		50	55 (111)
HfNbTaTiZr	[69], [70]	BCC	(9.9)	390	C	929		50	(111)
HfNbTiVZr	[57]	BCC + lm	(8.1)	388	C	1170	1463	30	128
HfNbTiZr	[71]	BCC	(8.4)		T	879	969	15	(92)
MoNbTaV	[72]	BCC	(10.7)	504	C	1525	2400	21	(187)
MoNbTaVW	[73]	BCC	(12.4)	536	C	1246	1270	2	(232)
MoNbTaW	[73]	BCC	(13.7)	454	C	1058	1211	2	(258)
MoNbTiV	[54]	BCC	(7.3)	441	C	1200		26	(170)
Mo0.3NbTiVZr	[74]	BCC	6.7		C	1289		42	
Mo0.5NbTiVZr	[74]	BCC	6.8		C	1473		32	
Mo0.7NbTiVZr	[74]	BCC	7.0		C	1706		32	
MoNbTiVZr	[74]	BCC	7.1		C	1779		32	
Mo1.3NbTiVZr	[74]	BCC	7.3		C	1496		30	
Mo1.5NbTiVZr	[74]	BCC	7.4		C	1603		20	
Mo1.7NbTiVZr	[74]	BCC	7.5		C	1645		15	
Mo2NbTiVZr	[74]	BCC	7.6		C	1765		12	
MoNbTiV0.25Zr	[75]	BCC	(7.3)		C	1776	3893	30	(153)
MoNbTiV0.50Zr	[75]	BCC	(7.2)		C	1647	3307	28	(152)
MoNbTiV0.75Zr	[75]	BCC	(7.2)		C	1708	3929	29	(150)
MoNbTiV1.0Zr	[75]	BCC	(7.1)		C	1786	3828	26	(149)
MoNbTiV1.5Zr	[75]	BCC	(7.1)		C	1735	3300	20	(147)
MoNbTiV2.0Zr	[75]	BCC	(7.0)		C	1538	3176	23	(146)
MoNbTiV3.0Zr	[75]	BCC	(6.9)		C	1418	2508	24	(143)
MoNbTiZr	[75]	BCC	(7.3)		C	1592	3450	34	(155)
NbTaTiV	[55]	BCC	(9.2)		C	1092			106 (134)
NbTaVW	[76]	BCC	(12.9)	492	C	1530		12	(208)
NbTaTiVW	[76]	BCC+HCP	(11.1)	447	C	1420		20	
NbTiV0.3Zr	[74]	BCC	6.5		C	866		45	
NbTiV0.3Mo0.1	[74]	BCC	6.6		C	932		45	
NbTiV0.3Mo0.3	[74]	BCC	6.8		C	1312		50	
NbTiV0.3Mo0.5	[74]	BCC	6.9		C	1301		43	
NbTiV0.3Mo0.7	[74]	BCC	7.1		C	1436		27	
NbTiV0.3Mo	[74]	BCC	7.3		C	1455		25	
NbTiV0.3Mo1.3	[74]	BCC	7.4		C	1603		20	
NbTiV0.3Mo1.5	[74]	BCC	7.5		C	1576		8	
NbTiVZr	[74]	BCC	6.5		C	1104		50	
NbTiVZr	[59]	BCC	(6.5)	335	C	1105		> 50	(104)
NbTiV2Zr	[59]	BCC	(6.4)	304	C	918		> 50	(109)
**Other HEAs and CCAs**									
CoCrCuFeNiTiVZr	[27]		(7.1)	680					(168)
CoCrFeMoNiTiVZr	[27]		(7.3)	850					(193)
CoCuFeNiTiVZr	[27]		(7.1)	630					
CoFeNiV	[77]	FCC	(7.8)	238					(187)
CoFeMo0.2NiV	[77]	FCC + Im	(8.0)	267					
CoFeMo0.4NiV	[77]	FCC + Im	(8.1)	402					
CoFeMo0.6NiV	[77]	FCC + Im	(8.2)	557					
CoFeMo0.8NiV	[77]	FCC + Im	(8.3)	606					
CoFeMoNiV	[77]	FCC + Im	(8.4)	625					
CoFeMoNi1.2V	[77]	FCC + Im	(8.4)	602					
CoFeMoNi1.4V	[77]	FCC + Im	(8.5)	538					
CoFeMoNi1.6V	[77]	FCC + Im	(8.5)	520					
CoFeMoNi1.8V	[77]	FCC + Im	(8.5)	510					
CoFeMoNi2V	[77]	FCC + Im	(8.5)	382					
CoFeMoNiTiVZr	[27]		(7.3)	790					
CuFeNiTiVZr	[27]		(6.8)	590					(142)
CoCrCuFeMnNiTiV	[78]	FCC + BCC + Im	(7.3)		C	1312	1312	< 1	74
Al11.1(CoCrCuFeMnNiTiV)88.9	[78]	FCC + BCC	(6.7)		C	1862	2431	< 1	164 (182)
Al20(CoCrCuFeMnNiTiV)80	[78]	BCC	(6.1)		C	1465	2016	2	190 (180)
Al40(CoCrCuFeMnNiTiV)60	[78]	BCC + Im	(5.1)		C	1461	1461	< 1	163
AlFeNiTiVZr	[27]	BCC	(5.9)	800					(132)
(CuMnNi)75Zn25	[79]	FCC	(8.3)	147	C	215		> 60	(169)
(CuMnNi)80Zn20	[79]	FCC	(8.3)	109	C	140		> 65	(171)
(CuMnNi)90Al10	[79]	FCC + Im	(8.1)	241	C	515		40	
(CuMnNi)90Sn10	[79]	FCC + Im	(8.3)	318	C	630		20	
(CuMnNi)95Al5	[79]	FCC	(8.3)	166	C	330		> 45	(174)
(CuMnNi)95Sn5	[79]	FCC + Im	(8.4)	205	C	380		> 63

**Table 2:** HEAs and CCAs for which mechanical tests are reported in literature as a function of temperature.

**Composition**	**Refs.**	**Phase**	***ρ* (g/cm**^**3**^**)**	***T* (°C)**	***σ**^**y**^***(MPa)**	***ε* (%)**
Al0.3NbTa0.8Ti1.4V0.2Zr1.3	[52]	BCC	7.8 (7.7)	25	1965	5
				800	678	> 50
				1000	166	> 50
Al0.3NbTaTi1.4Zr1.3	[52]	BCC	8.2 (8.1)	25	1965	5
				800	362	> 50
				1000	236	> 50
Al0.4Hf0.6NbTaTiZr	[52]	BCC	9 (9.1)	25	1841	10
				800	796	> 50
				1000	298	> 50
Al0.5CoCrCuFeNi	[80]	FCC	7.9 (7.6)	1000	150	
				25	388	
				300	411	
				500	421	
				700	426	
				900	230	
				1100	80	
Al0.5NbTa0.8Ti1.5V0.2Zr	[52]	BCC	7.4 (7.6)	25	2035	5
				800	796	> 50
				1000	220	> 50
Al2CoCrCuFeNi	[80]	BCC	6.7 (6.3)	1000	116	
				1100	79	
				25	1620	
				600	805	
				500	1120	
				700	567	
				900	214	
				800	302	
AlCoCrCuFeNi	[80]	FCC + BCC	7.4 (7.1)	1000	47	
				25	948	
				600	561	
				700	307	
				800	172	
				900	98	
AlCrMoNbTi	[81]	BCC	(6.6)	25		
				400	1080	2
				600	1060	3
				800	860	2
				1000	594	15
				1200	105	24
AlMo0.5NbTa0.5TiZr	[52]	BCC	7.4 (7.1)	25	2000	10
				800	1597	11
				1000	745	> 50
				1200	250	> 50
AlNb1.5Ta0.5Ti1.5Zr0.5	[52]	BCC	6.9 (6.8)	25	1280	4
				800	728	> 12
				1000	403	> 50
AlNbTiV	[56]	BCC	5.6 (5.5)	25	1020	5
				600	810	12
				800	685	50
				1000	158	50
CrHfNbTiZr	[57]	BCC + lm	(8.1)	25	1375	3
				300	1420	4
				500	1457	2
				700	1322	1
				900	1328	5
CrMo0.5NbTa0.5TiZr	[28]	BCC + Im	8.2 (8)	25	1595	5
				800	983	6
				1000	546	50
				1200	170	50
CrNbTiVZr	[59]	BCC + Im	6.6	25	1298	3
				600	1230	10
				800	615	> 50
				1000	259	> 50
CrNbTiZr	[59]	BCC + Im	6.7 (6.6)	25	1260	6
				600	1035	> 50
				800	300	> 50
				1000	115	> 50
HfMoNbTaTiZr	[63]	BCC	9.97 (9.95)	25	1512	12
				800	1007	23
				1000	814	30
				1200	556	30
HfMoNbTiZr	[64]	BCC	8.7	25	1575	9
				800	825	50
				1000	635	50
				1200	187	50
HfMoTaTiZr	[63]	BCC	10.24 (10.21)	25	1600	4
				800	1045	19
				1000	855	30
				1200	404	30
HfNbSi0.5TiV	[65]	BCC + lm	8.6 (7.8)	25	1399	11
				800	875	50
				1000	240	50
HfNbSi0.5TiVZr	[66]	BCC + lm	7.75 (7.5)	0	1540	17
				600	1252	50
				800	427	50
HfNbTaTiZr	[40]	BCC	9.9	25	929	50
				600	675	50
				800	535	50
				1000	295	50
				1200	92	50
				1400	790	50
HfNbTiVZr	[57]	BCC + lm	(8.1)	25	1170	30
				300	1120	30
				500	1253	38
				700	1140	30
				900	1157	40
MoNbTaVW	[73]	BCC	12.4	25	1246	2
				600	862	13
				800	846	17
				1000	842	19
				1200	735	8
				1400	656	40
				1600	477	40
MoNbTaW	[73]	BCC	13.8 (13.7)	25	1058	3
				600	561	40
				800	552	40
				1000	548	40
				1200	506	40
				1400	421	40
				1600	405	40
NbTiV2Zr	[59]	BCC	6.3 (6.4)	25	918	50
				600	571	50
				800	240	50
				1000	72	50
NbTiVZr	[59]	BCC	6.5	25	1105	50
				600	834	50
				800	187	50
				1000	58	50
